# Energy Based Logic Mining Analysis with Hopfield Neural Network for Recruitment Evaluation

**DOI:** 10.3390/e23010040

**Published:** 2020-12-30

**Authors:** Siti Zulaikha Mohd Jamaludin, Mohd Shareduwan Mohd Kasihmuddin, Ahmad Izani Md Ismail, Mohd. Asyraf Mansor, Md Faisal Md Basir

**Affiliations:** 1 School of Mathematical Sciences, Universiti Sains Malaysia, Penang 11800, Malaysia; szulaikha.szmj@usm.my (S.Z.M.J.); shareduwan@usm.my (M.S.M.K.); ahmad_izani@usm.my (A.I.M.I.); 2 School of Distance Education, Universiti Sains Malaysia, Penang 11800, Malaysia; 3 Department of Mathematical Sciences, Faculty of Science, Universiti Teknologi Malaysia, Bahru, Johor 81310, Malaysia; mfaisalmbasir@utm.my

**Keywords:** satisfiability representation, Hopfield neural network, logic mining, recruitment evaluation, economic well-being

## Abstract

An effective recruitment evaluation plays an important role in the success of companies, industries and institutions. In order to obtain insight on the relationship between factors contributing to systematic recruitment, the artificial neural network and logic mining approach can be adopted as a data extraction model. In this work, an energy based *k* satisfiability reverse analysis incorporating a Hopfield neural network is proposed to extract the relationship between the factors in an electronic (E) recruitment data set. The attributes of E recruitment data set are represented in the form of *k* satisfiability logical representation. We proposed the logical representation to 2-satisfiability and 3-satisfiability representation, which are regarded as a systematic logical representation. The E recruitment data set is obtained from an insurance agency in Malaysia, with the aim of extracting the relationship of dominant attributes that contribute to positive recruitment among the potential candidates. Thus, our approach is evaluated according to correctness, robustness and accuracy of the induced logic obtained, corresponding to the E recruitment data. According to the experimental simulations with different number of neurons, the findings indicated the effectiveness and robustness of energy based *k* satisfiability reverse analysis with Hopfield neural network in extracting the dominant attributes toward positive recruitment in the insurance agency in Malaysia.

## 1. Introduction

Systematic recruitment evaluation requires an optimal decision support system in ensuring the high-quality services by the insurance agents that will propel the success of the insurance companies in Malaysia. In order to hire the right insurance agent, several independent bodies such as Life Insurance Management Research Association (LIMRA) introduced various tests to screen potential candidates [1]. Corporations in insurance company or agencies are harnessing aggregated operational data to facilitate their recruitment activities based on the predictions as mentioned in [2,3]. Candidates are required to attend a pre-requisite seminar before they can advance to the next stage of the interview selection. Therefore, a comprehensive rule is needed to classify the attendance of the recruit. An insurance company need to expand their recruitment market by getting more candidates [4]. Consequently, the higher amount of attendance during this pre-requisite seminar will increase the chances for more agents to get contracted. The main challenge faced by recruitment personnel is low number of attendance record retention during pre-requisite seminar. In some cases, the number of candidates who does not committed to their attendance can reach up to 90% to 95% of total candidate. In other words, the company is losing resources to physically accommodate the wrong candidates. Therefore, selecting the right candidate based on their preliminary attributes will reduce work task and increase the effectiveness of the recruitment team. Conventional recruitment systems are generally based on machine learning, regression analysis and decision tree discovery [5]. The methods perform well in the classification with specific computations. However, an alternative approach is needed to extricate the relationships of the factors contributing to the positive or negative recruitment. The term positive recruitment in this case is the ability for the human resource (HR) to choose the right candidate that will attend the pre-requisite seminar. Hence, more comprehensive “advice” is required to assist the HR personnel to make an informed decision. This can be achieved by capitalizing artificial neural network (ANN) and logic mining method.

The advancement of data mining in recruitment has been growing due to the development of Industrial Revolution 4.0. Data mining is generally categorized as an association, clustering, classification and prediction based on any given data sets [6,7]. Ref. [4] proposed a data mining technique by integrating the decision tree in predicting the job performance and personnel selection. Thus, the knowledge extraction approach via decision tree produced an acceptable degree of accuracy. However, it would be difficult to analyze the behavior of the data from the attributes. In this context, behavior of the data can be defined as the pattern of the data that leads to the specific desired output. In addition, the application of machine learning approach such as support vector machine paradigm in predicting the risk in HR has been discussed in the work of [8]. The results were acceptable, but the method only focuses on the classification only. Thus, the real information from each of the attributes cannot be interpreted comprehensively. Ref. [9] extended the decision tree approaches over the k means clustering techniques in screening the job applicants to the industry. In relation to this, Ref. [10] utilized an adaptive selection model approach in recruitment. Both methods provided good results in term of error evaluations. In addition, the underlying behavior of the data set need to be extracted separately. Next, [11] applied the back-propagation network in forecasting management associate’s recruitment rates for different enterprises. In their work, the probability of the attributes is computed before being trained and tested by back propagation neural network to check the probability of a recruit to stay in that firm. A recent work by [12] has indicated the ability of the recurrent neural network (RNN) as the building block for ability-aware person job fit neural network (APJFNN) model in training an industrial data set in China. The proposed model recorded a better accuracy compared to the state-of-the-art approaches such as decision tree, linear regression and gradient boosting decision tree. Since most of the aforementioned data mining techniques integrate the statistical measures, an alternative method will be appropriate in facilitating the learning and testing phase of the recruitment data set.

Based on the recent theoretical developments of artificial neural network (ANN), have revealed the capability in various data mining tasks such as classification and clustering. For instance, successive geometric transformations model (SGTM) is a neural-like model as proposed by Tkachenko and Izonin [13], which being applied in [14] for the electric power consumption prediction for combined-type industrial areas. Izonin et al. [14] has successfully demonstrated the efficiency of SGTM as compared with the statistical regression analysis. In another development, Tkachenko et al. [15] further extended the work by proposing general regression neural network with successive geometric transformation model (GRNN-SGTM) ensemble. The work has increased the predictive capability based on the accuracy in missing Internet of Thing (IoT) data mining. Radial basis neural network (RBFNN) is a variant of multilayer feedforward ANN that can be explored in various application due to the forecasting capability in several works. Villca et al. [16] utilized a radial basis function neural network (RBFNN) in predicting the optimum chemical composition during mining processes especially in copper tailings flocculation processes. Mansor et al. [17] incorporates Boolean 2 satisfiability logical representation into RBFNN by obtaining the special parameters such as width and centre. The effective wind speed horizon has been forecasted with higher level of correctness as shown in the work of Madhiarasan [18]. Some works leveraged the Adaline neural network approach in various forecasting tasks such as in power filter optimization [19] and interior permanent magnet synchronous motor (IPMSM) parameter prediction [20]. Both work of Sujith and Padma [19] and Wang et al. [20] utilized an Adaline neural network as a classifier for the parameters involved in industrial control problem. The deep convolutional neural network (DCNN) is a variant of powerful ANN, with multi-layer hidden neurons that play important role for the data prediction. Li et al. [21] utilized the DCNN in assessing the remaining useful life (RUL) assessment and forecast to extract the insight on the maintenance factors for equipment and machineries in industry. Sun et al. [22] applied the DCNN in the city traffic flow management towards the intelligent transport system (ITS). Houidi et al. [23] proposed the DCNN in forecasting the appropriate pattern during the non-intrusive load monitoring. These works have been successful in prediction task based on the high accuracy values obtained after the simulations.

The Hopfield neural network (HNN) is regarded as one of the earliest ANN that imitate how the brain computes. HNN was proposed by Hopfield [24] to solve various optimization problems. HNN is a class of recurrent ANN without any hidden layer that demonstrates high-level learning behavior. This includes an effective learning and retrieval mechanism. An important property of HNN is the energy minimization of the neurons whenever the neuron changes state. Even though HNN has a simple structure (without hidden layer), HNN remains relevant to numerous field of studies such as optimization of ANN [25,26], bio-medical imaging [27], engineering [28], Mathematics [29], communication [30] and data mining [31]. An important property of HNN is the energy minimization of the neurons whenever the neuron changes state. Since the traditional HNN is prone to a few weaknesses such as lower neuron interpretability [32], logic programming was embedded to HNN as a single intelligent unit [33,34]. The work of logic programming in HNN capitalizes the effective logical rule being trained and retrieved by HNN with the aim to generate the solutions with the minimum energy. In particular, Sathasivam [35] introduced the Horn satisfiability (HORNSAT) in HNN with the dynamic neuron relaxation rates. It was observed that the proposed model obtained higher global minima ratio for the dynamic neuron relaxation as opposed to the constant relaxation rate. Kasihmuddin et al. [36] further developed the *k* satisfiability *(k*SAT) logic programming in HNN, with the priority given to improve the learning phase of the model via genetic algorithm. The work of [36] is a major breakthrough of *k*SAT as a systematic Boolean satisfiability logical representation, without any redundant structure. The simulation has confirmed the improvement of *k*SAT logic programming in HNN in attaining optimal final states that drives towards global minimum solutions as compared with [35]. Mansor and Sathasivam [37] formulated a variant of *k*SAT, known as 3 satisfiability (3SAT) logic programming, specifically as a representation of 3-dimensional logical structure. The systematic logical rule as 3SAT as coined in [37] can be seen to comply with the HNN as proven by the performance evaluation metrics such as global minima ratio, Hamming distance and computation (CPU) time. Velavan et al. [38] proposed mean field theory (MFT) by implementing Boltzmann machine and output squashing via hyperbolic activation function for Horn satisfiability logic programming in HNN. Theoretically, the work in [38] has slightly outperformed [35] even though the similar logic structure being utilized. Kasihmuddin et al. [39,40] extended the restricted maximum *k*-satisfiability (MAX*k*SAT) programming in HNN, where the emphasis was given to the unsatisfiable logical rule under different level of neuron complexities. Based on the study reported in [40], MAX*k*SAT logical rule performed optimally as compared to the Kernel Hopfield neural network (KHNN). The effectiveness of various logic programming in HNN has been proved in previously aforementioned works, which bring another perspective of representing the real data in the form of logical representation. In short, we need a well-established logical rule that can represents the behavior of the recruitment data.

Logic mining is a variant of data extraction process by leveraging the Boolean logic and ANN. Ref. [41] has proposed logic mining method in HNN by implementing reverse analysis method. The proposed logic mining technique is capable of extracting logical rule among neurons. The early work on reverse analysis method by incorporating Horn satisfiability logical rule in neural network was introduced by [42] in processing the customer demand from a supermarket via reverse analysis simulation. However, the existence of redundancy in the extracted logical representation was found to be non-systematic due to the lack of interpretability of the behavior of a particular real data set. Hence, a notable model known as *k* satisfiability (*k*SAT) in reverse analysis method has been specifically implemented in various applications. An efficient medical diagnosis of non-communicable diseases such as the Hepatitis, Diabetes and Cancer have implemented in Kasihmuddin et al. [43] by employing 2 satisfiability reverse analysis (2SATRA). The proposed logic mining technique has extracted the behavior or symptoms of the non-communicable diseases with more than 83% accuracy. Moreover, Kho et al. [44] has effectively developed 2SATRA method in extracting the optimal relationship between the strategies and gameplay in League of Legends (LoL), a variant of well-known electronic sport (e-sport). The extracted logic with the highest accuracy has been extracted that will benefit the e-sport coaches and players. In other development, Alway et al. [31] extracted the logical rule that represent the behaviour of the prices of the palm oil and other commodities by using 2SATRA and HNN. It was found that the systematic weight management has affected the optimal induced logic as a representation of the palm oil prices data set. These works have emphasized the logic mining in terms of systematic 2SAT representation, with a good capability in exploring the behaviour of the data. In addition, Zamri et al. [45] proposed 3 satisfiability reverse analysis (3SATRA) to extract the prioritized factor in order to grant or revoke employees resources applications in an International online shopping platform. The work in [45] has recorded 94% of accuracy, indicating the effectiveness of the logic mining approach over the conventional methods. Hence, the implementation of various *k* satisfiability (*k*SAT) in reverse analysis method in extracting the data set is still limited, especially in recruitment evaluation. Thus, in this research a novel Energy based *k* satisfiability reverse analysis method will be developed to extract the correct recruitment factors that lead to positive recruitment in an insurance company in Malaysia. By using the extracted logical rule, the recruitment personnel are expected to properly strategize their recruitment force and target the insurance agent with good quality. 

**Figure 1:**
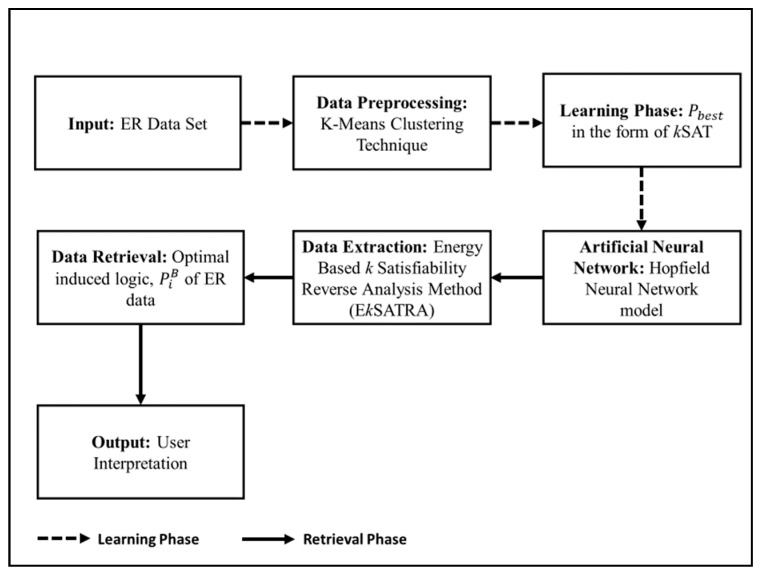
General implementation of the proposed model.

The contributions of this work are as follows: (a) to convert the E recruitment data set into systematic form, based on *k*SAT representation. (b) To propose the energy based *k* satisfiability reverse analysis method as an alternative approach in extracting the relationships between the factors or attributes that contribute to the positive recruitment based on E recruitment data obtained from an insurance agency in Malaysia. (c) To assess the capability and accuracy of two variants of the proposed method based on 2SAT and 3SAT logical representation as compared to Horn satisfiability in completing the E recruitment data extraction with different number of clauses. The performance evaluations metrics will be adopted to evaluate the effectiveness of both proposed method and logical representations as an alternative data extraction method to the E recruitment system. The general implementation of energy based *k* satisfiability reverse analysis method and HNN in extracting E recruitment (ER) data is illustrated in Figure 1.

This paper is organized as follows. Section 2 discusses the theoretical aspect of *k* satisfiability representation in general. Section 3 emphasizes the important implementation of *k* satisfiability representation in discrete Hopfield neural network by introducing the essential formulations used during the learning and testing phase of our model. Following this, Section 4 highlights the fundamental concepts of energy based *k* satisfiability reverse analysis method, as the data extraction paradigm for E recruitment evaluation data set. Section 5 presents the implementation of energy based *k* satisfiability reverse analysis method in extracting the insightful relationship among the attributes of E recruitment data set. Following that, Section 6 and Section 7 focus on the formulation of performance evaluation metrics and simulation setup involved in this work. Section 8 presents the results and discussion based on the performance of our proposed energy based *k* satisfiability reverse analysis method model in learning and testing using the E recruitment datasets. Section 9 reports concluding remarks of this work.

## 2. *k* Satisfiability Representation

*k* satisfiability (*k*SAT) is a logical representation that consist of strictly *k* variables per clause [36]. The properties of *k*SAT can be summarized as follows:

- A set of m logical variables, $x_{1},x_{2},\ldots ,x_{m}$. Each variable store a bipolar value of $x_{i}\in \left\{1,-1\right\}$ that exemplify TRUE and FALSE respectively.
- Each variable in $x_{i}$ can be set of literals where positive literal and negative literal is defined as $x_{m}$ and $\neg x_{m}$ respectively.
- Consist of a set of n distinct clauses, $C_{1},C_{2},C_{3},\ldots ,C_{n}$. Each $C_{i}$ is connected by logical AND (∧). Every *k* literals will be form a single $C_{i}$ and connected by logical OR (∨).

By using property (i) until (iii), we can define the explicit definition of *k*SAT formulation or $P_{kSAT}$:

(1) $$P_{kSAT}=\overset{n}{\underset{i=1}{\wedge }}C_{i}$$

This study considers k=2,3. Hence the clear definitions of $C_{i}^{\left(k\right)}$ for k=2 and k=3 are as follows:

(2) $$C_{i}^{\left(2\right)}=\overset{n}{\underset{j=1}{\vee }}\left(x_{ij},y_{ij}\right),\;k=2$$

(3) $$C_{i}^{\left(3\right)}=\overset{n}{\underset{j=1}{\vee }}\left(x_{ij},y_{ij},z_{ij}\right),\;k=3$$

where $C_{i}^{\left(2\right)}$ and $C_{i}^{\left(3\right)}$ are 2SAT and 3SAT clause respectively. Consider the formulation of $P_{kSAT}$ that consist of variable A, T,G,E,O and S where,¬ A signifies the negation of variable A.

The example of $P_{kSAT}$ for both k=2 and k=3 are:

(4) $$P_{2SAT}=\left(A\vee T\right)\wedge \left(G\vee Ε\right)\wedge \left(\neg O\vee S\right),\;k=2$$

(5) $$P_{3SAT}=\left(\neg T\vee S\vee \neg R\right)\wedge \left(A\vee C\vee G\right)\wedge \left(E\vee O\vee K\right),\;k=3$$

According to Equation (4), the possible assignment that leads to $P_{2SAT}=1$ is (A,T,E,O,G,S)=(1,1,1,1,1,1). In this case, the $P_{2SAT}$ is said to be satisfiable because all are $C_{i}^{\left(2\right)}=1$. $P_{kSAT}$ will read −1 if any of the clause in the formula is $C_{i}^{\left(2\right)}=-1$. Similar observation can be deduced in k=3. The formulation for both $P_{2SAT}$ formulation must be represented in conjunctive normal form (CNF) because the satisfiability nature of CNF can be conserved compared to other form such as disjunctive normal form (DNF). Equations (4) and (5) do not consider any redundant variables that may result in unsatisfiable nature of Equations (2) and (3). In addition, we only consider the assignment that leads to $P_{2SAT}=1$, interested reader may refer to [34,40] for $P_{2SAT}=-1$. In this paper, we represent the information of the datasets in the form of attributes. The attributes are defined as variables in $P_{2SAT}$ and become the symbolic rule for ANN. 

## 3. *k* Satisfiability in Discrete Hopfield Neural Network

Hopfield neural network (HNN) was popularized by J. J Hopfield in 1985 to solve various optimization problem [24]. Consider the conventional HNN model that consist of mutually connected neurons $S_{i}=\left(S_{1},S_{2},S_{3},\ldots ,S_{N}\right)$ where $S_{i}=\left\{-1,1\right\}$. All $S_{i}$ are assumed to be updated asynchronously according to the following equation:

(6) $$S_{i}\left(t\right)=\left\{ \begin{array}{l} \;\;\;\;1\;\;,\;\;\mathrm{if}\;\;\sum_{j}^{N}W_{ij}S_{j}\left(t\right)+\beta \geq 0\\ -1\;\;,\;\;Otherwise \end{array}\right.$$

where $W_{ij}$ is the synaptic matrix that connects neuron *j* to *i* (of the total of N interconnected neurons) with pre-determined bias β. In HNN, the state produced by Equation (6) signifies the possible solution for any given optimization problem. Note that, the final state obtained will be further verified by using final energy analysis where the aim of HNN is to find the final state the corresponding to global minimum energy. By capitalizing the updating property in Equation (6), $P_{kSAT}$ is reported to be compatible to the structure of HNN [46]. In this work, $P_{kSAT}$ is embedded as a symbolic instruction to the HNN by assigning each neuron with a set of m variables. For any given $P_{kSAT}$ that is embedded into HNN, the cost function of $E_{P_{kSAT}}$ for $P_{kSAT}$ is defined as follows [36]:

(7) $$E_{P_{kSAT}}=\sum_{i=1}^{n}\prod_{j=1}^{m}Z_{ij}$$

where n and m in Equation (7) signify the number of clause and variables in $P_{kSAT}$ respectively. Note that, the inconsistencies of $\neg P_{kSAT}$ are given as:

(8) $$Z_{ij}=\left\{ \begin{array}{l} \frac{1}{2}\left(1-S_{X}\right),\;\;if\;\;\neg \;X\\ \frac{1}{2}\left(1+S_{X}\right),\;\;otherwise \end{array}\right.$$

where $S_{X}$ is the neuron for variable X. The cost function is $E_{P_{kSAT}}=0$ when all $C_{i}$ in Equation (2) reads $C_{i}=1$. Note that, $E_{P_{kSAT}}\neq 0$ if at least one $C_{i}=-1$. The primary aim of the hybrid HNN is to minimize the value of $E_{P_{kSAT}}$ as the number $P_{kSAT}$ clause increase. The updating rule $h_{i}\left(t\right)$ to the final state of HNN that incorporates the proposed $P_{kSAT}$ is given as follows:

(9) $$h_{i}\left(t\right)=\sum_{i=1,\;i\neq j}^{n}W_{ij}^{\left(2\right)}S_{j}+W_{i}^{\left(1\right)},\;k=2$$

(10) $$h_{i}\left(t\right)=\sum_{i=1,\;i\neq j\neq k}^{n}W_{ijk}^{\left(3\right)}S_{j}S_{k}\;\;\;+\sum_{i=1,\;i\neq j}^{n}W_{ij}^{\left(2\right)}S_{j}+W_{i}^{\left(1\right)},\;\;k=3$$

(11) $$S\left(t\right)=\left\{ \begin{array}{l} \;\;\;\;1\;\;,\;\;h\left(t\right)\geq 0\\ -1\;\;,\;\;h\left(t\right)<0 \end{array}\right.$$

where $W_{ijk}^{\left(3\right)}$, $W_{ij}^{\left(2\right)}$ and $W_{i}^{\left(1\right)}$ are third (three neuron connections per clause), second (two neuron connections per clause) and first order (one neuron connection per clause) synaptic weight respectively. The synaptic weight of $P_{kSAT}$ in HNN is symmetrical and zero self-connection:

(12) $$W_{ijk}^{\left(3\right)}=W_{ikj}^{\left(3\right)}=W_{jki}^{\left(3\right)}=W_{kji}^{\left(3\right)}=W_{jik}^{\left(3\right)}=W_{kij}^{\left(3\right)}$$

(13) $$W_{ij}^{\left(2\right)}=W_{ji}^{\left(2\right)}$$

(14) $$W_{iii}^{\left(2\right)}=W_{jjj}^{\left(2\right)}=W_{kkk}^{\left(2\right)}=W_{ii}^{\left(2\right)}=W_{kk}^{\left(2\right)}=0$$

In practice, optimal value for (12)–(14) can be obtained by comparing Equation (7) with the following final energy:

(15) $$H_{P_{kSAT}}=-\frac{1}{2}\sum_{i=1,i\neq j}^{n}\sum_{j=1,i\neq j}^{n}W_{ij}^{\left(2\right)}S_{i}S_{j}-\sum_{i=1,i\neq j}^{n}W_{i}^{\;\left(1\right)}S_{j}\;\;,\;k=2$$

(16) $$H_{P_{kSAT}}=-\frac{1}{3}\sum_{i=1,i\neq j\neq k}^{n}\sum_{j=1,i\neq j\neq k}^{n}\sum_{k=1,i\neq j\neq k}^{n}W_{ijk}^{\left(3\right)}S_{i}S_{j}\;-\frac{1}{2}\sum_{i=1,i\neq j}^{n}\sum_{j=1,i\neq j}^{n}W_{ij}^{\left(2\right)}S_{i}S_{j}\;-\sum_{i=1,i\neq j}^{n}W_{i}^{\;\left(1\right)}S_{j}\;\;,\;k=3$$

The step of comparing the final energy with the cost function is known as Wan Abdullah method [33]. Note that, the final energy $H_{P_{kSAT}}$ in Equations (15) and (16) can be obtained by considering the asynchronous update of the neuron state for specific $P_{kSAT}$. The quality of the neuron states based on the energy function as shown in Equation (15) for k=2 and Equation (16) is specialized for k=3. $H_{P_{kSAT}}$ can be further updated by computing the differences in the energy produced by local field. Consider the neuron update for $P_{kSAT}$ at time t. The final energy that $P_{kSAT}$ is given as follows:

(17) $$H_{P_{kSAT}}\left(t\right)=-\frac{1}{2}\sum_{i=1,i\neq j}^{n}\sum_{j=1,i\neq j}^{n}W_{ij}^{\left(2\right)}S_{i}S_{j}-\sum_{i=1,i\neq j}^{n}W_{i}^{\left(1\right)}S_{i}$$

(18) $$H_{P_{kSAT}}\left(t+1\right)=-\frac{1}{2}\overset{n}{\underset{i=1,i\neq j}{\sum }}\overset{n}{\underset{j=1,i\neq j}{\sum }}W_{ij}^{\left(2\right)}S_{i}^{q}S_{j}-\overset{n}{\underset{i=1,i\neq j}{\sum }}W_{i}^{\left(1\right)}S_{i}^{q}$$

$H_{P_{kSAT}}\left(t\right)$ refers to the energy before being updated by q that store $P_{kSAT}$ patterns. Thus, the updated $H_{P_{kSAT}}\left(t+1\right)$ will verify the final state produced after the learning and retrieval phase. The differences of energy level are given as:

(19) $$\Delta H_{P_{kSAT}}=H_{P_{kSAT}}\left(t\right)-H_{P_{kSAT}}\left(t+1\right)$$

By substituting Equations (17) and (18),

$$\Delta H_{P_{kSAT}}=-\frac{1}{2}\left(S_{i}-S_{i}^{q}\right)\left(\overset{n}{\underset{i=1,i\neq j}{\sum }}W_{ij}^{\left(2\right)}S_{j}+W_{i}^{\left(1\right)}\right)$$

By simplifying it and being compared with Equation (10),

(20) $$\Delta H_{P_{kSAT}}=-\frac{1}{2}\left(S_{i}-S_{i}^{q}\right)h_{i}\left(t\right)$$

**Figure A1:**
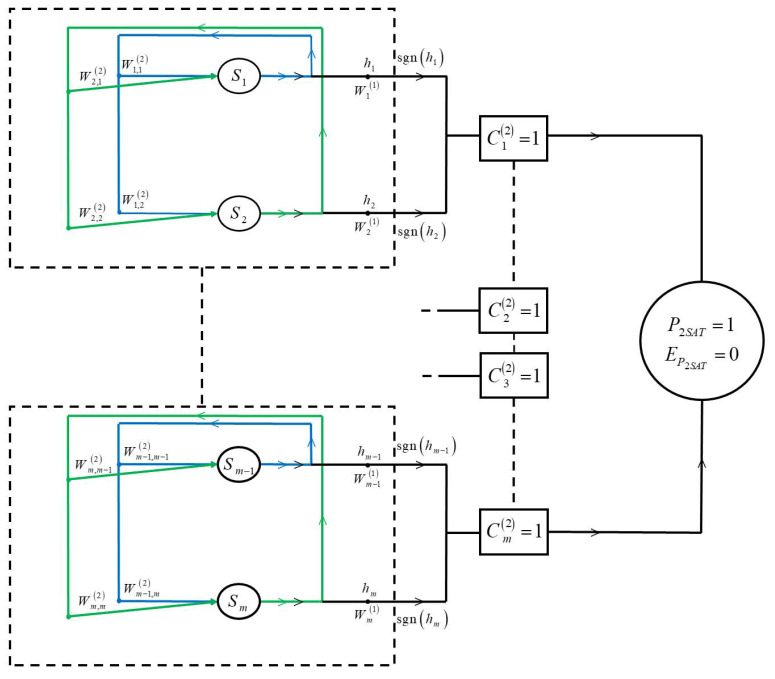
The Schematic Diagram of HNN-*k*SAT.

Based on Equation (20), it can be concluded HNN will reach a state whereby the energy cannot be reduced further. The similar states will indicate the optimized final states that leads to $\Delta H_{P_{KSAT}}=0$. This equilibrium will ensure the early validation of our final state of the neurons. Figure A1 shows the implementation of $P_{kSAT}$ in HNN. Another interesting note about the implementation of $P_{kSAT}$ in HNN is the ability of the model to calculate the minimum energy supposed to be $H_{P_{kSAT}}^{\min}$. Minimum energy supposed to be can be defined as the absolute minimum energy achieved during retrieval phase. $H_{P_{kSAT}}^{\min}$ can be obtained by using the following formula:

(21) $$H_{P_{kSAT}}^{\min}=\left\{ \begin{array}{l} \;\;\;\;\;\;-\frac{\theta +2\eta }{4}\;\;\;\;\;\;\;\;\;\;,k=2\\ -\frac{2\theta +4\eta +\gamma }{8}\;\;\;\;\;,k=3 \end{array}\right.$$

where $\gamma =n\left(C_{i}^{\left(3\right)}\right)$, $\theta =n\left(C_{i}^{\;\left(2\right)}\right)$ and $\eta =n\left(C_{i}^{\;\left(1\right)}\right)$ that corresponds to $P_{kSAT}$. The newly formulated $H_{P_{kSAT}}^{\min}$ has reduced the computational burden to the existing calculation. The previous $H_{P_{kSAT}}^{\min}$ computation in [35,36,37,38] requires the randomized states and energy function, which already being simplified as in Equation (21) for $P_{kSAT}$. By taking into account the value obtained from Equations (15) and (16), the final state of HNN is considered optimal if the network satisfies the following condition:

(22) $$\left|H_{P_{kSAT}}-H_{P_{kSAT}}^{\min}\right|\leq \partial$$

where ∂ is a tolerance value pre-determined by the user. Worth mentioning that the final state of the Equations (15) and (16) will be converted into induced logic. Several studies implemented other type of logical rule such as HORNSAT [35] and improved HNN such as mean field theory (MFT) [38] will be converted into induced logic during logic extraction. In this work, the final states that correspond to the global minimum energy will be the focus during the retrieval phase of HNN model. The idea of energy based HNN will be extended in improving the existing logic mining by energy verification for each of the induced logic extracted by the model. Hence, the newly proposed energy based *k* satisfiability reverse analysis will be hybridized with HNN in extracting the behavior of the dataset. 

## 4. Energy Based *k* Satisfiability Reverse Analysis Method (E*k*SATRA)

One of the limitations of the standalone HNN in knowledge extraction is the interpretation of the output. Usually, the output of the conventional HNN can be interpreted in terms of bipolar state which requires expensive output checking. Hence, logic mining connects propositional logical rule (HNN-2SAT or HNN-3SAT) with knowledge extraction by implementing ANN as a learning system. The pattern of the dataset can be extracted and represented via logical rule obtained by HNN. This section formulates an improved reverse analysis method, which is energy based *k* satisfiability reverse analysis method of E*k*SATRA. E*k*SATRA is structurally different from the previous *k*SATRA because only logical rule that comply with Equation (22) will be converted into induced logic. The following algorithm illustrate the implementation of E*k*SATRA:

**Step** **1:**  Consider n number of attributes $\left(S_{1},S_{2},S_{3},S_{4},\ldots ,S_{n}\right)$ of the datasets. Convert all binary dataset into bipolar representation:
  (23) $$S_{c}=\left\{ \begin{array}{l} \;\;\;\;1\;\;,\;\;\;S_{c}=1\\ -1\;\;,\;\;otherwise \end{array}\right.$$

The state of $S_{i}$ is defined based on the neural network conventions where 1 is considered as TRUE and –1 as FALSE. $S_{i}$ and $S_{j}$, i≠j are the collection of neuron that represent the $C_{1}^{\left(2\right)}$. $S_{c}$, $S_{v}$ and $S_{b}$, c≠v≠b is the case for $C_{1}^{\left(3\right)}$. Note that, $C_{1}^{\left(2\right)}$ and $C_{1}^{\left(3\right)}$ are clauses for $P_{2SAT}$ and $P_{3SAT}$ respectively. Hence, the collection of $C_{1}^{\left(k\right)}$ that leads to positive outcome of the learning data or $P_{i}^{\;l}=1$ will be segregated.

****Step** **2:****  Calculate the collection of $C_{i}^{\left(k\right)}$ that frequently leads to $P_{i}^{\;l}=1$. The optimum logic $P_{best}$ of the dataset is given as follows:
  (24) $$P_{best}=\max\left[n\left(P_{i}^{\;l}=1\right)\right]$$

Note that, if $P_{best}$ must be in the form of Boolean algebra [44] which corresponds to Equations (2) and (3). Derive the cost function $E_{P_{best}}$ by using Equation (7).

**Step** **3:**  Find the state of $S_{i}$ that corresponds to $E_{P_{best}}=0$. Hence by comparing $E_{P_{best}}$ with $H_{P_{best}}$, the synaptic weight of HNN-*k*SAT will be obtained in [33].
**Step** **4:**  By using Equations (9) and (10), obtain the final state of the HNN-*k*SAT. The variable assignment $S_{i}^{induced}$ for each $C_{i}^{\left(k\right)}$ will be based on the following condition:
  (25) $$S_{i}^{induced}=\left\{ \begin{array}{l} \;\;\;\;A\;\;\;\;\;\;,\;\;\;S_{A}^{induced}=1\\ \neg \;A\;\;\;\;\;,\;\;\;S_{A}^{induced}=-1 \end{array}\right.$$

Note that the variable assignment will formulate the induced logic $P_{i}^{\;B}$.

**Step** **5:**  Calculate the final energy that corresponds to the value of $S_{i}^{induced}$ by using Equations (15) and (16). Verify the energy by using the following condition:
  (26) $$P_{i}^{B}=\left\{ \begin{array}{l} P_{i}^{B}\;\;\;\;,\;\left|H_{P_{i}^{B}}-H_{P_{i}^{B}}^{\min}\right|\leq \partial \\ 0\;\;\;\;\;\;\;\;\;\;\;\;,otherwise \end{array}\right.$$

The threshold value ∂ is predefined by the user (usually $10^{-\;4}$). According to Equation (26) only $P_{i}^{B}$ that achieve global minimum energy that will proceed to the testing phase.

**Step** **6:**  Construct the induced logic $P_{i}^{B}$ from Equation (26). By using test data from the dataset, verify whether $P_{i}^{B}=P_{i}^{\;test}$. Note that, $P_{i}^{test}$ is the test data provided by the user.

The verified induced logic, $P_{i}^{B}$ by the energy function will be extracted at the end of the E*k*SATRA, indicating the correct $P_{kSAT}$ logical representation of the behavior of the data set. This is different as compared to the existing work in [44] which focuses on the unverified induced logic by the energy function. This innovation is important in deciding the quality of the $P_{i}^{B}$ produced at the end of the retrieval phase.

## 5. E*k*SATRA in E Recruitment Data Set

E recruitment (ER) data is a data set obtained from an insurance agency in Malaysia [47]. The data set contains 155 candidate’s information such as age, past occupation, education background, origin, online texting, criminal record, keep in view list, citizenship status, and source of candidate. Previously, the insurance agency attempts to analyze the data by using the statistical approach such as logistic regression. Even though the results were acceptable, the behavior of the ER dataset will remain difficult to observe. Thus, the recruiter requires a comprehensive approach so that the behavior of the data set can be extracted systematically even though a new set of data will be added in the future. Hence, the logic mining approach via *k*SATRA will provide a solid logical rule as a representation of the ER data set to the recruiter from the insurance agency in Malaysia. This will be utilized to generate a logical rule to represent the behavior of the data.

In this work, there are different attributes being entrenched in 2SATRA and 3SATRA respectively. The aim of ER data is to extract the logical rule that explain the behavior of the candidates. This logical rule will determine their attendance during pre-requisite seminar. ER data will be divided into learning data and testing data. In learning data set, {Attend,Not Attend} will be converted into bipolar representation {1,−1} respectively. Each attribute of the candidate will be represented in terms of neuron in *k*SATRA. Hence there will be a total of *k* neurons per clause will be considered in this data set. In this regard, *k*SATRA contains collection of neuron formula that leads to $P_{i}^{\;l}=Attend(P_{i}^{\;l}=1)$ or $P_{i}^{\;l}=Not\;Attend(P_{i}^{\;l}=-1)$. For example, one of the candidates $P_{i}^{\;l}$ is able to communicate via online texting (WhatsApp or Facebook Messenger), is aged less than 25 years old, has no past occupation, and an education background higher than SPM (Sijil Pelajaran Malaysia; the Malaysia high school diploma), does not originate from Kota Kinabalu (headquarters of the company) and the resume was sent through email. The $P_{i}^{\;l}$ has the following neuron interpretation:

(27) $$\begin{array}{r} \;\;\;\;\;\;\;\;\;\;\;\;\;\;\;\;\;Online\;\;Texting\left(W\right)\;\;\;=\;\;\;\;\;1\\ \;\;\;\;\;\;\;\;\;\;\;\;\;\;\;\;\;\;\;\;\;\;\;\;\;\;\;\;\;\;\;\;\;\;\;\;\;\;\;\;Age\left(A\right)\;\;\;=-1\\ \;\;\;\;\;\;\;\;\;\;\;\;\;\;\;Past\;Occupation\left(G\right)\;\;=-1\\ Education\;\;Background\left(E\right)\;\;=\;\;\;\;\;1\\ \;\;\;\;\;\;\;\;\;\;\;\;\;\;\;\;\;\;\;\;\;\;\;\;\;\;\;\;\;\;\;\;\;\;\;\;Origin\left(O\right)\;\;=-1\\ \;\;\;\;\;\;\;\;\;\;\;\;\;\;\;\;\;\;\;\;\;\;\;\;\;\;\;\;\;\;\;\;\;\;\;\;Source\left(S\right)\;\;=\;\;\;\;\;\;1 \end{array}$$

By converting the above attributes into logical rule, $P_{i}^{\;l}$ will reads

(28) $$P_{i}^{\;l}=\left(W\vee \neg A\right)\wedge \left(\neg G\vee E\right)\wedge \left(\neg O\vee S\right)$$

where $P_{i}^{\;l}=1$ for candidate i=1. In other word, E*k*SATRA “learned” that the candidate attended $\left(P_{i}^{\;l}=1\right)$ the pre-requisite seminar if they satisfy any of the neuron interpretation in Equation (28). The above steps will be repeated to find the rest of the $P_{i}^{\;l}$ where i=1,2,3,…N. Hence the network will obtain the initial $P_{best}$ and will be embedded to HNN. In order to derive the correct synaptic weight, the network will find the correct interpretation that leads to $E_{P_{best}}=0$. During retrieval phase of E*k*SATRA, HNN will retrieve the induced logic that optimally explain the relationship of the attributes for the candidates. One of the possible induced logical rule $P_{i}^{B}$ is as follows:

(29) $$\begin{array}{l} P_{i}^{B}=A\leftarrow W\\ \;\;\;\;\;\;\;\;\;\;\;\;G\leftarrow E\\ \;\;\;\;\;\;\;\;\;\;\;\;O\leftarrow S \end{array}$$

**Figure 2:**
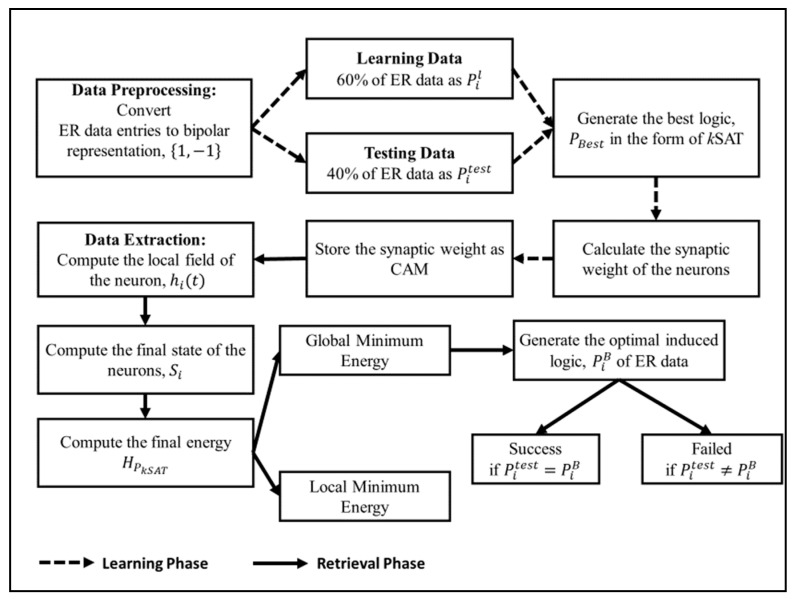
The block diagram of energy based *k* satisfiability reverse analysis method (E*k*SATRA) implementation in E-recruitment (ER) dataset.

Equation (29) is the logical rule that generalize the behavior of the whole candidates in HNN. The symbol (←) represents implication of variables that leads to $P_{i}^{B}$. Similar to 2SAT, the logic extraction method will be applied to 3SAT representation. The information from the logical rule helps the recruitment team to analyze and generalize the performance of the candidate based on simple logical induction. By using the induced logic $P_{i}^{B}$, recruitment team can classify the attendance of the candidate to pre-requisite seminar. This induced logic $P_{i}^{B}$ will assist the recruitment team by creating more effective strategies to address only significant attribute(s) that reduce the number of absentees for company’s event. Less attention will be given to unimportant attributes which will reduce cost and management time. The full implementation of E*k*SATRA in ER data set extraction is shown as a block diagram in Figure 2. 

Figure 2 shows that the E*k*SATRA can be divided into learning and retrieval phase, before obtaining the logical representation that can be used in explaining the relationship and behaviour of ER data set.

## 6. Performance Evaluation Metric

In this section, a total of four performance evaluation indicators are deployed to analyze the effectiveness of our E*k*SATRA model in extracting important logical rule in ER datasets. Note that, all the proposed metrics evaluate the performance of the learning and testing phase. Since the integrated ANN in the proposed E*k*SATRA is HNN, the proposed metric solely indicates the performance of the retrieved neuron state that contribute to $P_{i}^{B}$. During the learning phase, the performance of the *k*SAT representation that governs the network will be evaluated based on the following fitness equation:

(30) $$f_{i}=\sum_{i=1}^{NC}C_{i}$$

NC is the number of clause for any given $P_{i}^{B}$. According to Equation (30), $C_{i}$ is defined as follows:

(31) $$C_{i}=\left\{ \begin{array}{l} \;1\;\;\;\;True\\ 0\;\;\;False \end{array}\right.$$

Note that, as $f_{i}$ approaching to NC, the value of $E_{P_{kSAT}}$ will be minimized to zero. By using the obtained $f_{i}$, the performance of the learning phase will be evaluated based on root mean square error (RMSE), mean absolute error (MAE), mean absolute percentage error (MAPE) and computation time (CPU).

### 6.1. Root Mean Square Error

Root mean square error (RMSE) [36] is a standard error estimator that widely been used in predictions and classifications. During the learning phase, RMSE measures the deviation of the error between the current value $f_{i}$ and NC with respect to mean ${\bar{f}}_{i}$. The Learning_RMSE is based on the following equation

(32) $$\mathrm{LEARNING}\text{\_}\mathrm{RMSE}=\sum_{i=1}^{n}\sqrt{\frac{1}{n}{\left(f_{\max}-f_{i}\right)}^{2}}$$

where $f_{\max}=NC$ and $f_{\max}$ is dependent to the number of *k*SAT clauses. During retrieval phase, RMSE measures the deviation of the error between the current $P_{i}^{B}$ with the state of the $P_{i}^{test}$.

(33) $$\mathrm{TESTING}\text{\_}\mathrm{RMSE}=\sum_{i=1}^{n}\sqrt{\frac{1}{n}{\left(P_{i}^{\;test}-P_{i}^{\;B}\right)}^{2}}$$

Note that, lower value of Learning_RMSE signifies the compatibility of *k*SAT in E*k*SATRA, likewise lower value of Testing_RMSE signifies the small error deviation of the proposed $P_{i}^{B}$ with respect to the $P_{i}^{test}$.

### 6.2. Mean Absolute Error

The mean absolute error (MAE) [44] is another type of error that evaluate the straightforward difference between the expected value and the current value. During the learning phase, MAE measures the absolute difference between the current value $f_{i}$ and NC. The Learning_MAE is based on the following equation:

(34) $$LEARNING\_MAE=\sum_{i=1}^{n}\frac{1}{n}\;\left|P_{i}^{\;test}-P_{i}^{\;B}\right|$$

where $f_{\max}=NC$ and $f_{\max}$ is dependent to the number of *k*SAT clauses. During retrieval phase, MAE measures the deviation of the error between the current $P_{i}^{B}$ with the state of the $P_{i}^{\;test}$.

(35) $$TESTING\_MAE=\sum_{i=1}^{n}\frac{1}{n}\;\left|P_{i}^{\;test}-P_{i}^{\;B}\right|$$

Note that, lower value of Learning_MAE signifies the compatibility of *k*SAT in E*k*SATRA, likewise lower value of Testing_MAE signifies the small error deviation of the proposed $P_{i}^{B}$ with respect to the $P_{i}^{test}$. 

### 6.3. Mean Absolute Percentage Error 

Mean absolute percentage error (MAPE) [44] measures the size of the error in form of percentage terms. During the learning phase, MAPE measures the percentage difference between the current value $f_{i}$ and NC. The Learning_MAPE is based on the following equation:

(36) $$LEARNING\_MAPE=\sum_{i=1}^{n}\frac{100}{n}\;\frac{\left|f_{\max}-f_{i}\right|}{\left|f_{i}\right|}$$

where $f_{\max}=NC$ and $f_{\max}$ is dependent to the number of *k*SAT clauses. During retrieval phase, MAE measures the deviation of the error between the current $P_{i}^{B}$ with the state of the $P_{i}^{test}$.

(37) $$TESTING\_MAPE=\sum_{i=1}^{n}\frac{100}{n}\;\frac{\left|P_{i}^{B}-P_{i}^{\;test}\right|}{\left|P_{i}^{\;test}\right|}$$

Note that, lower value of Learning_MAPE signifies the compatibility of *k*SAT in E*k*SATRA, likewise lower value of Testing_MAPE signifies the small error deviation of the proposed $P_{i}^{B}$ with respect to the $P_{i}^{test}$. In other word, high value of Learning_MAPE will result to more $E_{P_{kSAT}}\neq 0$ which is reported to affect the quality of the retrieval phase. Hence, inconsistent $P_{i}^{B}$ will result in lower accuracy of the proposed logic mining.

### 6.4. CPU Time

CPU time is defined as a time acquired by a model to complete the learning phase and retrieval phase. In the perspective of learning phase, CPU time is calculated from the $f_{i}$ into $f_{\max}$ whereas CPU time for retrieval phase is calculated from initial neuron state $S_{i}\left(t\right)$ until $S_{i}\left(t+1\right)$ where $S_{i}\left(t+1\right)=S_{i}^{induced}$. Hence, the simple definition for CPU_Time is as follows:

(38) CPU_Time=Learning_Time+Retrieval_Time

CPU_Time has been utilized in several papers [34,40] for examining the complexity of the proposed HNN-*k*SAT model.

## 7. Simulation Setup

The simulation is designed to evaluate the capability of E*k*SATRA in extracting the relationship between the ER data attributes in terms of optimal $P_{i}^{B}$. In this study, 60% of the candidate data sets in ERHNN will be used as $P_{i}^{\;l}$ and 40% will be utilized as $P_{i}^{test}$ of the learning phase of E*k*SATRA. The learning to testing data ratio, 3:2 is chosen to comply with the work of Kho et al. [44]. All HNN models were implemented in Dev C++ Version 5.11 in Windows 10 (Intel Core i3, 1.7 GHz processor) with different complexities. In order to avoid possible bad sector, the simulation is conducted in a similar device. According to [36], the threshold CPU time for both learning phase and testing phase is set as one day (24 h). If E*k*SATRA exceeds the proposed threshold CPU time, the $P_{i}^{B}$ will not compared with $P_{i}^{test}$. Regarding on the neuron variation issue during the retrieval phase, clausal noise (CN) has been added to avoid possible overfitting. The equation relating to NC and CN is as follows:

(39) $$NC=NC_{attribute}+CN$$

where $NC_{attribute}$ is the candidate’s attribute in HNN. 

In this study, the setting $NC_{attribute}=1$ considering the number of clause in each $P_{kSAT}$ corresponds to the value of $NC_{attribute}$. In practice, NC has a linear relationship to the number of CN and $P_{3SAT}$ is expected to experience more CN compared $P_{2SAT}$. In terms of logical rule that will be embedded inside HNN, the existing work of Sathasivam and Abdullah [41] that implemented HORNSAT in their proposed reverse analysis method. In this study [41], the embedded HORNSAT logical rule must consist at most one positive literal for any proposed clause in the formulation. The proposed HORNSAT embedded in HNN has been improved by the work of Velavan et al. [38]. In this work [38], they proposed the combination of hyperbolic activation function and Boltzmann machine to reduce unnecessary neuron oscillation during the retrieval phase. Both of these proposed models were considered the only existing logic mining in the literature. The existing method were abbreviated as HNN-HORNSAT and HNN-MFTHORNSAT. Table 1 illustrates the parameter setup for HNN-*k*SAT models: 

The important parameters such as the neuron combinations, number of learning, number of trial and neuron string should be set as 100 to comply with the work of Kasihmuddin et al. [39]. Neuron combination can be defined as a number of possible input combination input during the simulation. Number of learning is the learning iteration required for the proposed method to achieve $E_{P_{kSAT}}=0$ during the learning phase. Number of trials is the number of retrieved $P_{i}^{B}$ for each neuron combination. 

The optimal neuron combination is essential, as large number of neuron combination will increase the dimension of the searching space of the solution, resulting in the computational burden. In addition, if we set the small neuron combinations, the solution will lead to local minimum solutions. According to [37], hyperbolic tangent activation function (HTAF) was chosen due to the differentiability of the function and the capability to establish non-linear relationship among the neuron connections. Based on ER data set, there is no missing value, indicating that the complete data will be processed by our proposed method.

## 8. Result and Discussion

The performance of the simulated program with different complexities for HNN-*k*SAT models will be evaluated with the existing models HNN-HORNSAT [35] and HNN-MFTHORNSAT [38] in terms of root mean square error (RMSE), mean absolute error (MAE), mean absolute percentage error (MAPE), accuracy and CPU time.

**Figure 3:**
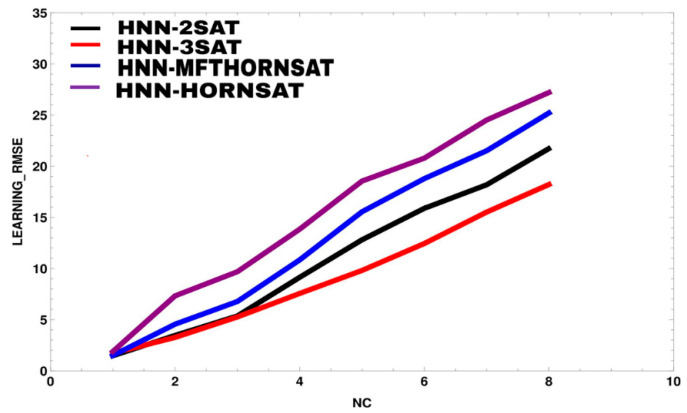
Learning root mean square error (RMSE) for all HNN-k satisfiability (kSAT) models.

**Figure 4:**
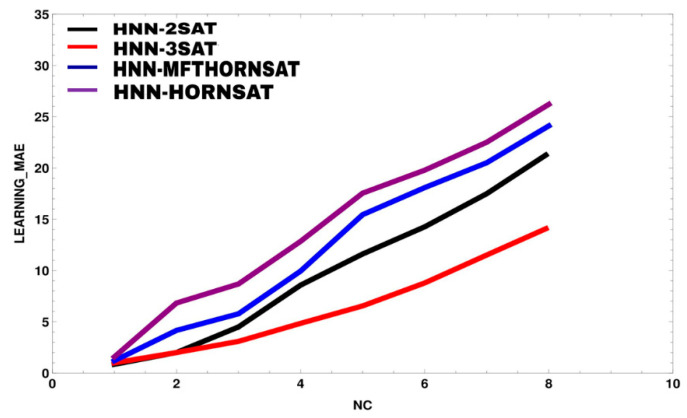
Learning mean absolute error (MAE) for all HNN-*k*SAT models.

Figure 3 and Figure 4 illustrate root mean square error (RMSE) and mean absolute error (MAE) of HNN models during learning phase. It is worth noting that this analysis only proposes strictly two or three literals per clause. The data has successfully embedded into the network and forming a learnable *k*SAT logic. The comparison has been made between the proposed models, HNN-2SAT and HNN-3SAT with the existing methods, namely HNN-HORNSAT [35] and HNN-MFTHORNSAT [38]. As seen in Figure 2, HNN-*k*SAT with NC=2 until NC=8 has the best performance in terms of RMSE compared to HNN-HORNSAT and HNN-MFTHORNSAT. HNN-*k*SAT utilizes logical inconsistencies help E*k*SATRA to derive the optimum synaptic weight for HNN. Optimal synaptic weight is a building block for optimum $P_{i}^{B}$. The RMSE result from Figure 2 has been supported by the value of MAE in Figure 3.

Similar to Figure 3, HNN-*k*SAT with NC=1 has the best performance in terms of MAE. It can be observed that MAE for NC=1 is equal to 0.85 compared with NC=4 that recorded 8.571425 for HNN-2SAT. As the number of CN increased, learning phase of E*k*SATRA will be much convoluted because HNN-*k*SAT is required to find the consistent interpretation for $P_{best}$. In this case, learning phase of E*k*SATRA for both HNN-2SAT and HNN-3SAT trapped in trial and error solution that leads to RMSE and MAE accumulation. In contrast, the learning phase in HNN-HORNSAT and HNN-MFTHORNSAT were computationally expensive as more iterations needed leading to higher RMSE and MAE values compared to HNN-2SAT and HNN-3SAT. All in all, E*k*SATRA contributes in generating the best logic to represent the relationship between each instance and the verdict of HNN.

Table 2 manifests the MAPE obtained by HNN-2SAT, HNN-3SAT, HNN-HORNSAT and HNN-MFTHORNSAT during learning HNN. The value of MAPE produced by the four models is always less than 100%. Hence, error produced in every iteration for HNN-2SAT and HNN-3SAT during learning phase will increased as NC increases. However, the MAPE recorded by HNN-HORNSAT and HNN-MFTHORNSAT were to some extent higher than the proposed models. At NC=7, the value of MAPE in HNN-3SAT is approximately 55% times bigger than NC=8 because for the network to converge into full fitness (learning completed), more iterations needed. Therefore, the similar trend can be seen in HNN-2SAT as the complexity increases. Thus, HNN-2SAT and HNN-3SAT work optimally in learning the HNN entrenched to the network before being stored into content addressable memory. The complete learning process will ensure the network to generate the best logic to represent the characteristic of the HNN. Furthermore, it will be deployed during learning by using the remaining 40% of the data entries.

Table 3 displays the CPU Time results for the HNN models respectively. To assess the robustness of the models in logic mining, CPU time is recorded for the learning and retrieval phase of HNN. According to Table 3, less CPU Time are required to complete one execution of learning and testing for ER when the number of NC deployed is less. As it stands, HNN-2SAT and HNN-3SAT models require substantial amount of time to complete the learning when the complexity is higher. Overall, the HNN remains competent in minimizing the *k*SAT inconsistencies and compute the global solution within the acceptable CPU time. Hence, the CPU Time for HNN-3SAT is consistently higher than HNN-2SAT due to the more instances need to be processed during the learning and testing phase of HNN. However, the CPU time recorded for the existing methods, HNN-HORNSAT and HNN-MFTHORNSAT were apparently higher due to more iterations needed in generating the best logic for the HNN.

Table 4 shows the respective testing error recorded for both models during testing the HNN. Thus, the testing RMSE, MAE, accuracy and MAPE recorded for HNN-2SAT, HNN-3SAT, HNN-HORNSAT, and HNN-MFTHORNSAT were consistently similar for each of NC=1 until NC=8. Hence, this demonstrates the capability of our proposed network, E*k*SATRA and HNN-*k*SAT in generating the best logic, $P_{best}$ during the learning phase that contributes to a very minimum error during testing phase. The learning mechanism in E*k*SATRA in extracting the best logic to map the relationship of the attributes in HNN is acceptable according to performance evaluation metrics recorded during simulation. According to the accuracy recorded by each model, the proposed model achieved 63.30% positive recruitment outcome with HNN-2SAT and 85.00% for HNN-3SAT. According to Table 5, candidate in HNN-3SAT will give a negative result (not attend) if the candidate has the following conditions:

- ¬W: Cannot be reached via online texting.
- ¬ S: The resume was not sent through company’s email.
- ¬R: Has history of criminal report.
- ¬A: Aged less than 25 Years Old.
- ¬C: Non-Malaysan citizenship.
- ¬G: No past occupation.
- ¬E: The highest education is SPM (Malaysia High School Diploma).
- ¬O: Live outside Kota Kinabalu (state capital and headquarters of the company).
- ¬K: Not in “keep in view” list.

In another development, candidate in HNN-2SAT will give a negative result (not attend) if the candidate has the following conditions:

- ¬W: Cannot be reached via online texting.
- ¬A: Aged less than 25 Years Old.
- ¬G: No past occupation.
- ¬E: The highest education is SPM (Malaysia High School Diploma).
- ¬O: Live outside Kota Kinabalu (state capital and headquarters of the company).
- ¬S: The resume was not sent through company’s email.

The existing works on logic mining in HNN (HNN-HORNSAT and HNN-MFTHORNSAT) are unable to achieve at least 60% of the positive recruitment outcome. To sum up, HNN-3SAT incorporated with E*k*SATRA is the best model in learning and testing HNN due to lower values of RMSE, MAE, MAPE and the highest accuracy for logical rule. Hence, the logical rule obtained from HNN-3SAT will benefit the recruitment team in identifying the relationship between the candidates’ attributes and their eligibility to attend the pre-requisite seminar.

## 9. Conclusions

In conclusion, the findings have indicated the significant improvement of *k*SAT representation, logic mining technique and HNN in extracting the behavior of the real data set. Regarding on the non-optimal logical representation in the standard reverse analysis method in [41], quoting [43] that the flexible logical rule will make a tremendous impact in processing the ER data set in more systematic form. In this paper, we have successfully transformed the ER data set into optimal logical representation in the form of *k*SAT representation to best represent the relationship of ER data set. In addition, we have applied E*k*SATRA as an alternative approach in extracting the relationships between the attributes correspond to the positive recruitment of ER data set of an insurance company in Malaysia. Collectively, the proposed model, HNN-*k*SAT has explicitly produced the induce logic from the learned data with better accuracy as compared with HNN-MFTHORNSAT and HNN-HORNSAT. Apart from that, the effectiveness of *k*SAT in optimally representing the attributes of HNN is due to the simplicity in the structure of the logical representation. Hence, the relationship of the attributes in the ER data set has been extracted fruitfully with lower error evaluations and better accuracy. In order to counter the limitations, this research can be further developed in refining the learning phase of E*k*SATRA by employing the robust learning algorithms from the swarm-based metaheuristic to the evolutionary searching algorithm. Ultimately, the improved E*k*SATRA also can be extended to evaluate the retention rate of the insurance agents and in finding the significant factors in elevating the sales production.

## Figures and Tables

**Table 1:** List of parameters in Hopfield neural network (HNN) model.

Parameter	Value
Neuron Combination	100
Number of Learning	100
Number of Trial	100
Neuron String	100
Logical Rule (Differ for each model)	$P_{2SAT}$ [46] $P_{3SAT}$ [37] $P_{HORNSAT}$ [35] $P_{HORNSAT}$(MFT) [38]
Threshold CPU time	24 h
Threshold Energy (∂)	$10^{-4}$
Initial Neuron State	Random
Activation Function	Hyperbolic Tangent Activation Function
Clausal Noise (CN)	1≤CN≤10

**Table 2:** Learning mean absolute percentage error (MAPE) for all HNN models.

*NC*	HNN-HORNSAT	HNN-2SAT	HNN-3SAT	HNN-MFTHORNSAT
1	63.4500	28.3000	33.3331	55.4435
2	78.7860	33.3000	33.3334	67.7778
3	84.3566	50.0000	34.3333	76.0000
4	90.7400	71.4000	40.3761	84.5200
5	91.9260	91.4100	43.6514	91.7507
6	93.0052	92.6800	48.8182	92.9224
7	94.4528	92.8367	53.8935	93.0055
8	63.4500	28.3000	33.3331	55.4435

**Table 3:** Computation (CPU) time(s) for the HNN model.

*NC*	HNN-HORNSAT	HNN-2SAT	HNN-3SAT	HNN-MFTHORNSAT
1	1.8350	0.1963	0.3643	0.8232
2	2.9006	0.2733	0.6835	2.0679
3	5.6323	0.8780	1.3707	4.3258
4	12.0040	1.5076	2.2358	10.1566
5	18.9292	3.6846	6.7422	16.4340
6	25.1675	6.9666	11.4600	22.5552
7	32.0050	9.4050	14.2815	26.9081
8	48.0088	12.5590	26.9580	35.6528

**Table 4:** Testing error for all HNN models.

Model	Testing RMSE	Testing MAE	Testing MAPE	Accuracy (%)
HNN-HORNSAT	3.6140	0.4670	1.4583	53.30
HNN-2SAT	2.9210	0.3709	0.9649	63.30
HNN-3SAT	1.1430	0.1451	0.2941	85.00
HNN-MFTHORNSAT	3.3560	0.4333	1.2745	56.70

**Table 5:** The $P_{best}$ for all HNN models.

Model	$P_{best}$
HNN-HORNSAT	$\begin{array}{l} C_{1}=W\vee \neg \;A\vee \neg \;G\\ C_{2}=E\vee \neg O\\ C_{3}=S \end{array}$
HNN-2SAT	$\begin{array}{l} C_{1}=W\vee A\;\;\;\;\;\;\;\;\;\;\;\;\;\;\;\;\\ C_{2}=G\vee E\;\;\;\;\;\;\;\;\;\;\\ C_{3}=\neg O\vee S \end{array}$
HNN-3SAT	$\begin{array}{l} C_{1}=\neg W\vee S\vee \neg R\\ C_{2}=A\vee C\vee G\\ C_{3}=E\vee O\vee K \end{array}$
HNN-MFTHORNSAT	$\begin{array}{l} C_{1}=W\vee A\vee \neg G\;\;\;\;\\ C_{2}=E\vee \neg O\\ C_{3}=S \end{array}$

## Appendix A

The schematic diagram for HNN models for k=2 is given as follows:

## References

[1] Lasim P., Fernando M.S.C., Pupat N. (2016). Raising awareness of career goals of insurance agents: A case study of Choomthong 24K26, AIA Company. ABAC ODI J. Vision. Action. Outcome.

[2] Brockett P.L., Cooper W.W., Golden L.L., Xi X. (1997). A case study in applying neural networks to predicting insolvency for property and casualty insurers. J. Oper. Res. Soc..

[3] Delmater R., Monte H. (2001). Data Mining Explained: A Manager’s Guide to Customer-Centric Business Intelligence.

[4] Chien C.F., Chen L.F. (2008). Data mining to improve personnel selection and enhance human capital: A case study in high-technology industry. Expert Syst. Appl..

[5] Kohavi R., Quinlan J.R., Willi K., Jan M.Z. (2002). Data mining tasks and methods: Classification: Decision-tree discovery. Handbook of Data Mining and Knowledge Discovery.

[6] Osojnik A., Panov P., Džeroski S. (2016). Modeling dynamical systems with data streammining. Comput. Sci. Inf. Syst..

[7] Han J., Kamber M., Pei J. (2001). Data Mining: Concepts and Techniques.

[8] Li W., Xu S., Meng W. A Risk Prediction Model of Construction Enterprise Human Resources based on Support Vector Machine. Proceedings of the Second International Conference Intelligent Computation Technology and Automation, 2009 (ICICTA’09).

[9] Sivaram N., Ramar K. (2010). Applicability of clustering and classification algorithms for recruitment data mining. Int. J. Comput. Appl..

[10] Shehu M.A., Saeed F. (2016). An adaptive personnel selection model for recruitment using domain-driven data mining. J. Theor. Appl. Inf. Technol..

[11] Wang K.Y., Shun H.Y. (2016). Applying back propagation neural networks in the prediction of management associate work retention for small and medium enterprises. Univers. J. Manag..

[12] Qin C., Zhu H., Xu T., Zhu C., Jiang L., Chen E., Xiong H. Enhancing Person-Job Fit for Talent Recruitment: An Ability-Aware Neural Network Approach. Proceedings of the 41st International ACM SIGIR Conference on Research & Development in Information Retrieval.

[13] Tkachenko R., Izonin I. Model and principles for the implementation of neural-like structures based on geometric data transformations. Proceedings of the International Conference on Computer Science, Engineering and Education Applications.

[14] Izonin I., Tkachenko R., Kryvinska N., Tkachenko P. Multiple Linear Regression based on Coefficients Identification using Non-Iterative SGTM Neural-Like Structure. Proceedings of the International Work-Conference on Artificial Neural Networks.

[15] Tkachenko R., Izonin I., Kryvinska N., Dronyuk I., Zub K. (2020). An approach towards increasing prediction accuracy for the recovery of missing IoT data based on the GRNN-SGTM ensemble. Sensors.

[16] Villca G., Arias D., Jeldres R., Pánico A., Rivas M., Cisternas L.A. (2020). Use of radial basis function network to predict optimum calcium and magnesium levels in seawater and application of pretreated seawater by biomineralization as crucial tools to improve copper tailings flocculation. Minerals.

[17] Mansor M., Jamaludin S.Z.M., Kasihmuddin M.S.M., Alzaeemi S.A., Basir M.F.M., Sathasivam S. (2020). Systematic boolean satisfiability programming in radial basis function neural network. Processes.

[18] Madhiarasan M. (2020). Accurate prediction of different forecast horizons wind speed using a recursive radial basis function neural network. Prot. Control Mod. Power Syst..

[19] Sujith M., Padma S. (2020). Optimization of harmonics with active power filter based on ADALINE neural network. Microprocess. Microsyst..

[20] Wang L., Tan G., Meng J. (2019). Research on model predictive control of IPMSM based on adaline neural network parameter identification. Energies.

[21] Li H., Zhao W., Zhang Y., Zio E. (2020). Remaining useful life prediction using multi-scale deep convolutional neural network. Appl. Soft Comput..

[22] Sun S., Wu H., Xiang L. (2020). City-wide traffic flow forecasting using a deep convolutional neural network. Sensors.

[23] Houidi S., Fourer D., Auger F. (2020). On the use of concentrated time–frequency representations as input to a deep convolutional neural network: Application to non intrusive load monitoring. Entropy.

[24] Hopfield J.J., Tank D.W. (1985). “Neural” computation of decisions in optimization problems. Biol. Cybern..

[25] Kobayashi M. (2020). Diagonal rotor Hopfield neural networks. Neurocomputing.

[26] Ba S., Xia D., Gibbons E.M. (2020). Model identification and strategy application for Solid Oxide Fuel Cell using Rotor Hopfield Neural Network based on a novel optimization method. Int. J. Hydrog. Energy.

[27] Njitacke Z.T., Isaac S.D., Nestor T., Kengne J. (2020). Window of multistability and its control in a simple 3D Hopfield neural network: Application to biomedical image encryption. Neural Comput. Appl..

[28] Cai F., Kumar S., Van Vaerenbergh T., Sheng X., Liu R., Li C., Liu Z., Foltin M., Yu S., Xia Q. (2020). Power-efficient combinatorial optimization using intrinsic noise in memristor Hopfield neural networks. Nat. Electron..

[29] Tavares C.A., Santos T.M., Lemes N.H., dos Santos J.P., Ferreira J.C., Braga J.P. (2020). Solving ill-posed problems faster using fractional-order Hopfield neural network. J. Comput. Appl. Math..

[30] Yang H., Wang B., Yao Q., Yu A., Zhang J. (2019). Efficient hybrid multi-faults location based on hopfield neural network in 5G coexisting radio and optical wireless networks. IEEE Trans. Cogn. Commun. Netw..

[31] Alway A., Zamri N.E., Kasihmuddin M.S.M., Mansor M.A., Sathasivam S. (2020). Palm Oil Trend Analysis via Logic Mining with Discrete Hopfield Neural Network. Pertanika J. Sci. Technol..

[32] Gee A.H., Aiyer S.V., Prager R.W. (1993). An analytical framework for optimizing neural networks. Neural Netw..

[33] Abdullah W.A.T.W. (1992). Logic programming on a neural network. Int. J. Intell. Syst..

[34] Mansor M.A., Kasihmuddin M.S.M., Sathasivam S. (2017). Robust artificial immune system in the Hopfield network for maximum k-satisfiability. Int. J. Interact. Multimed. Artif. Intell..

[35] Sathasivam S. (2010). Upgrading logic programming in Hopfield network. Sains Malays..

[36] Kasihmuddin M.S.M., Mansor M.A., Basir M.F.M., Sathasivam S. (2019). Discrete mutation Hopfield Neural Network in propositional satisfiability. Mathematics.

[37] Mansor M.A., Sathasivam S. (2016). Accelerating activation function for 3-satisfiability logic programming. Int. J. Intell. Syst. Appl..

[38] Velavan M., Yahya Z.R., Halif M.N.A., Sathasivam S. (2016). Mean field theory in doing logic programming using Hopfield Network. Mod. Appl. Sci..

[39] Kasihmuddin M.S.B.M., Mansor M.A.B., Sathasivam S. (2016). Genetic algorithm for restricted maximum k-satisfiability in the Hopfield Network. Int. J. Interact. Multimed. Artif. Intell..

[40] Kasihmuddin M.S.M., Mansor M.A., Sathasivam S. (2018). Discrete Hopfield Neural Network in restricted maximum k-satisfiability logic programming. Sains Malays..

[41] Sathasivam S., Abdullah W.A.T.W. (2011). Logic mining in neural network: Reverse analysis method. Computing.

[42] Sathasivam S. (2012). Applying Knowledge Reasoning Techniques in Neural Networks. Aust. J. Basic Appl. Sci..

[43] Kasihmuddin M.S.M., Mansor M.A., Jamaludin S.Z.M., Sathasivam S. (2020). Systematic satisfiability programming in Hopfield Neural Network-A hybrid system for medical screening. Commun. Comput. Appl. Math..

[44] Kho L.C., Kasihmuddin M.S.M., Mansor M.A., Sathasivam S. (2020). Logic mining in league of legends. Pertanika J. Sci. Technol..

[45] Zamri N.E., Mansor M.A., Kasihmuddin M.S.M., Alway A., Jamaludin M.S.Z., Alzaeemi S.A. (2020). Amazon Employees Resources Access Data Extraction via Clonal Selection Algorithm and Logic Mining Approach. Entropy.

[46] Kasihmuddin M.S.M., Mansor M.A., Sathasivam S. (2020). Hybrid Genetic Algorithm in the Hopfield Network for Logic Satisfiability Problem. Pertanika J. Sci. Technol..

[47] Lee F.T. (2018). Monthly COP Report.

